# Cell-type specificity of lung cancer associated with low-dose soil heavy metal contamination in Taiwan: An ecological study

**DOI:** 10.1186/1471-2458-13-330

**Published:** 2013-04-10

**Authors:** Hsien-Hung Huang, Jing-Yang Huang, Chia-Chi Lung, Chih-Lung Wu, Chien-Chang Ho, Yi-Hua Sun, Pei-Chieh Ko, Shih-Yung Su, Shih-Chang Chen, Yung-Po Liaw

**Affiliations:** 1 Jen-Ai Hospital, Taichung City, 41265, Taiwan; 2 Department of Public Health and Institute of Public Health, Chung Shan Medical University, No. 110, Sec. 1, Chien-Kuo N. Road, Taichung City, 40201, Taiwan; 3 Department of Family and Community Medicine, Chung Shan Medical University Hospital, Taichung City, 40201, Taiwan; 4 School of Medicine and Institute of Medicine, Chung Shan Medical University, Taichung City, 40201, Taiwan; 5 Department of Orthopaedic Surgery, Chung Shan Medical University Hospital, Taichung City, 40201, Taiwan; 6 Department of Health and Leisure Management, Yuanpei University, Hsinchu City, 30015, Taiwan; 7 Department of Dentistry, Chung Shan Medical University, Taichung City, 40201, Taiwan; 8 Department of Leisure Industry and Health Promotion, National Ilan University, Yilan Country, 26047, Taiwan

**Keywords:** Lung cancer, Adenocarcinoma, Squamous cell carcinoma, Soil heavy metal, Age-standardized incidence rate

## Abstract

**Background:**

Numerous studies have examined the association between heavy metal contamination (including arsenic [As], cadmium [Cd], chromium [Cr], copper [Cu], mercury [Hg], nickel [Ni], lead [Pb], and zinc [Zn]) and lung cancer. However, data from previous studies on pathological cell types are limited, particularly regarding exposure to low-dose soil heavy metal contamination. The purpose of this study was to explore the association between soil heavy metal contamination and lung cancer incidence by specific cell type in Taiwan.

**Methods:**

We conducted an ecological study and calculated the annual averages of eight soil heavy metals (i.e., As, Cd, Cr, Cu, Hg, Ni, Pb, and Zn) by using data from the Taiwan Environmental Protection Administration from1982 to 1986. The age-standardized incidence rates of lung cancer according to two major pathological types (adenocarcinoma [AC] and squamous cell carcinoma [SCC]) were obtained from the National Cancer Registry Program conducted in Taiwan from 2001 to 2005. A geographical information system was used to plot the maps of soil heavy metal concentration and lung cancer incidence rates. Poisson regression models were used to obtain the adjusted relative ratios (RR) and 95% confidence intervals (CI) for the lung cancer incidence associated with soil heavy metals.

**Results:**

For males, the trend test for lung SCC incidence caused by exposure to Cr, Cu, Hg, Ni, and Zn showed a statistically significant dose–response relationship. However, for lung AC, only Cu and Ni had a significant dose–response relationship. As for females, those achieving a statistically significant dose–response relationship for the trend test were Cr (P = 0.02), Ni (P = 0.02), and Zn (P= 0.02) for lung SCC, and Cu (P < 0.01) and Zn (P = 0.02) for lung AC.

**Conclusion:**

The current study suggests that a dose–response relationship exists between low-dose soil heavy metal concentration and lung cancer occurrence by specific cell-type; however, the relevant mechanism should be explored further.

## Background

Residents living in areas where soil heavy metal concentration is elevated are prone to exposure to air, drinking water, and food with elevated heavy metal concentrations [1-11]. For example, Romero et al. [8] showed that the concentrations of copper (Cu) and zinc (Zn) in human serum were related to those in the soil and to the eating habits of local residents. The concentration of lead (Pb) in the blood of local adults is also related to that in the soil of the relevant area [9]. Cao et al. [10] reported that an area with Cu-contaminated water could cause both irrigated soil and rice to have significantly higher Cu concentrations. Cu concentration in human serum may increase as humans ingest water or food with a high Cu concentration [11]. These studies have shown that heavy metals in soil may affect the heavy metal concentration in human serum, thus exposing human tissues and organs to the metals. This is hypothesized to increase the risk of cancer incidence.

Several previous studies addressing the relationship between heavy metals and lung cancers have focused on occupational exposure of factory workers to heavy metals such as cadmium (Cd), chromium (Cr), and nickel (Ni) [12-15]; however, only a few studies have addressed Pb and lung cancer. Exposure to Pb can affect the central neuropathy and reproductive system [16,17]. One study has suggested the age-standardized lung cancer mortality rate of mercury (Hg) miners is higher than that of the general population [18]. Another study indicated that the lung cancer incidence rate of workers exposed to Hg vapor in chloralkali plants is higher than that of the general population [19]. Díez et al. [20] found that the Cu concentration or Cu/Zn ratio in the serum of lung cancer patients was higher than that of the control group. Zowczak et al. [21] also found that the Cu concentration in the serum of many cancer patients was higher than that of the control group. Coyle et al. [22] reported that the Zn discharge amount was positively related to the lung cancer incidence rate in Texas, USA.

In epidemiology, disease mapping may be used to explore disease variations and generate the hypothesis of an association between disease and environmental factors. John Snow investigated cholera in London by using the disease geographic variation to find the cause of cholera related to drinking water [23]. In 2005, a Japanese study used disease mapping to suggest that colon cancer and breast cancer were related to socioeconomic indicators within the studied areas [24]. Another study in the United States determined that the spatial variation patterns of smoking prevalence and lung cancer were similar [25] when comparing the temporal and geographic variations of lung cancer incidence for both white males and females from 1950 to 1994, as well as the smoking prevalence for both males and females in 1985.

To date, few studies have discussed the relationship between lung cancer incidence and soil heavy metal contamination, especially for specific cell types. Thus, this study used a geographical information system (GIS) and Poisson regression to explore the association between soil heavy metal contamination and lung cancer incidence by specific cell type.

## Methods

According to the 2003 Cancer Registry Annual Report from the Bureau of Health Promotion, Taiwan [26], 26.49% and 34.73% of lung cancer cases among males were reported as lung AC and SCC, respectively (12.06% for small-cell carcinoma); among females, 9.50% and 61.84% of lung cancer cases were reported as lung AC and SCC, respectively (3.51% for small-cell carcinoma). Compared with small-cell carcinoma, lung AC and SCC together apparently comprise the majority of all types of lung cancer in Taiwan. Therefore, this study focused on lung AC and SCC instead of other types. Lung AC and SCC cancer incidence data, restricted to patients older than 30 in 354 townships, were obtained from the National Cancer Registry Program (NCRP) operated by the Taiwanese government and collected for the cancer incidence cases from January 1, 2001 to December 31, 2005. We excluded patients younger than 30 because the characteristics of early-onset lung cancer are thought to be different from those of late-onset lung cancer. Both clinical and pathological diagnoses were coded using the ninth revision of the International Classification of Diseases for Oncology (ICD-O), based on ICD-O codes 80503, 81402, 81403, 81413, 81433, 82113, 82503, 82513, 82523, 82553, 82603, 83103, 83233, 84803, 84813, 84903, and 85003 for lung AC, and codes 80523, 80702, 80703, 80713, 80723, 80733, 80743, 80763, 80823, 80833, and 80843 for lung SCC. The population dataset was from the Taiwan-Fukien Demographic Fact Book issued by the Taiwan Ministry of the Interior from 2001 to 2005. The age ranges of the12 population groups were 30–34, 35–39, 40–44, 45–49, 50–54, 55–59, 60–64, 65–69, 70–74, 75–79, 80–84, and above 85.

Data for eight heavy metals (i.e., arsenic (As), Cd, Cr, Pb, Ni, Hg, Cu, and Ni) in the soil of 283 townships were collected during 1982 and 1986 from the Taiwan Environmental Protection Administration (TEPA). The data for surface soil (0–15 cm) were the results of the first-phase of a national heavy metal concentration survey conducted by the TEPA during 1982–1986 [27]. The survey was conducted in a large sample area of 1.16 million hectares of farmland soil in Taiwan, in the unit grid of 1,600 hectares, encompassing arrange of 283 townships. This study calculated the Spearman correlation coefficient for the concentrations of eight soil heavy metals in 1982–1986, and used GIS to explore the geographic variation of soil heavy metal concentration.

GIS software was also used to plot the map of lung cancer incidence. First, the age-standardized incidence rates (ASIRs) for two major cell-type-specific pathologies of lung cancer, AC and SCC, were calculated for both sexes in 354 townships across Taiwan in 2001–2005. These rates were sorted in descending order and then compared with the national means. The statistically significant differences in standardized incidence rates are represented by the seven colors, at which red denotes the highest 10% and a significantly high ASIR for the 354 townships; purple denotes not the highest 10% but significantly high; orange denotes the highest 10% but not significantly high; green denotes within 10-90% but not significantly different; grey denotes the lowest 10% but not significantly different; yellow denotes not the lowest 10% and significantly low, and white denotes the lowest 10% and significantly low.

Data were analyzed using SAS9.13 (SAS Institute, Cary, NC, USA). This study applied a Poisson regression to analyze the association between soil heavy metal concentrations of the 283 townships during 1982–1986 and the cell-type-specific pathologies of lung cancer incidence during 2001–2005. Regarding the quartiles of soil heavy metal concentration in 1982–1986, the first quartile group of the heavy metal concentration was treated as a reference group; the relative risk (RR) and 95% confidence interval (CI) of lung cancer in 2001–2005 were derived for both sexes by using Poisson regression models. In the Poisson regression models, lung cancer incidence cases were considered to be the dependent variable, which corresponds with the Poisson distribution with parameter μ. The independent variables in the model include dummy variables, established according to the quartiles of heavy metal concentration, age, and seven other heavy metals. A value of P <0.05 was considered statistically significant in all analyses.

## Results

**Figure 1:**
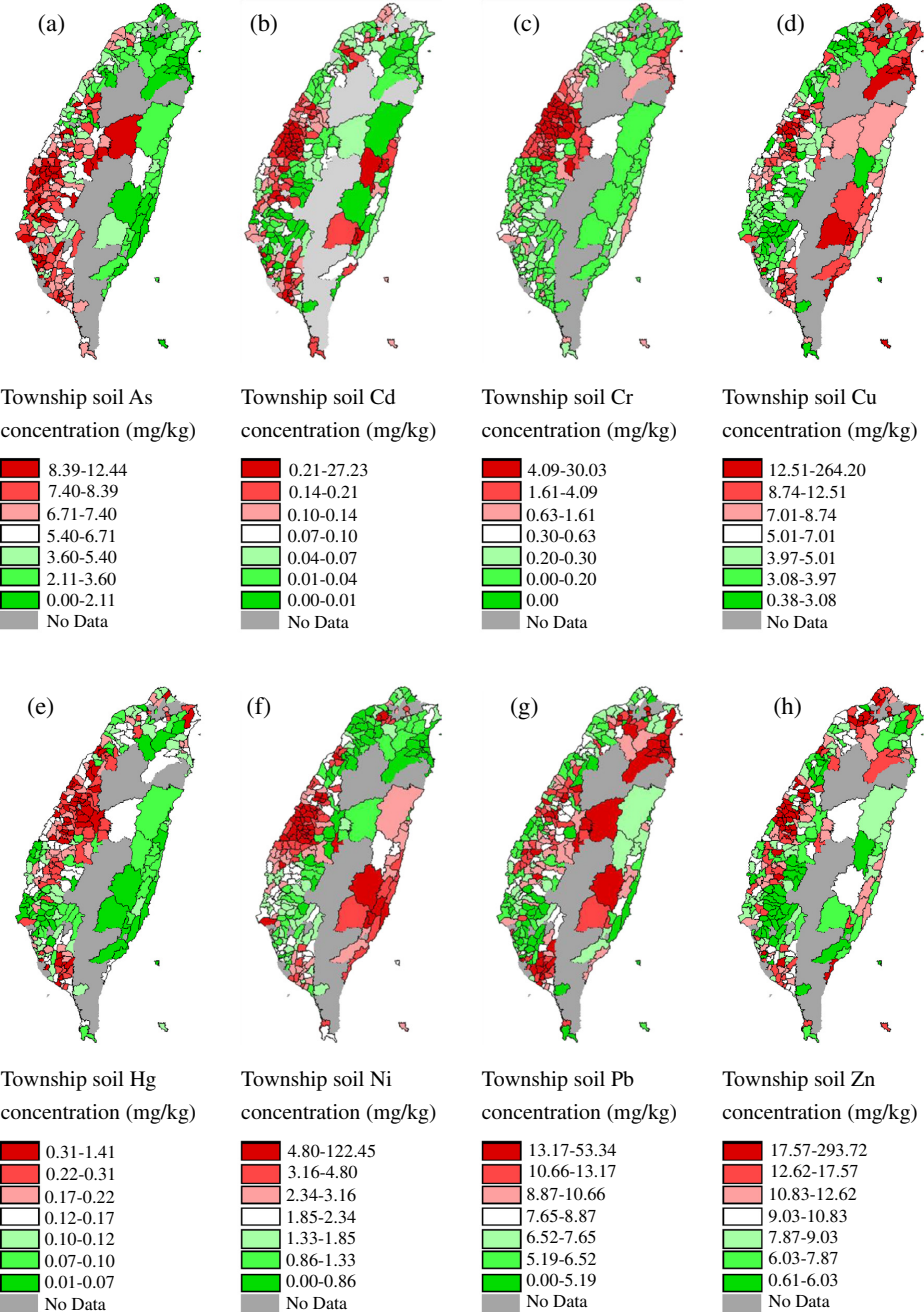
**Spatial distributions of township soil heavy metal concentration (mg/kg) during 1982-1986 in Taiwan.** Detailed legend: Spatial distributions of township soil heavy metal concentration (mg/kg) during 1982–1986 in Taiwan. (**a**) As, (**b**) Cd, (**c**) Cr, (**d**) Cu, (**e**) Hg, (**f**) Ni, (**g**) Pb, (**h**) Zn.

Figure 1 shows the distribution of the average concentrations of As (Figure 1a), Cd (Figure 1b), Cr (Figure 1c), Cu (Figure 1d), Hg (Figure 1e), Ni (Figure 1f), Pb (Figure 1g), and Zn (Figure 1h) in soil collected from the target townships during 1982–1986. The As concentration is higher in the southwestern coastal areas, and the Cd concentration is sporadically higher in northern and central Taiwan. Townships having higher Cr concentrations are in western and northeastern coastal areas. Cu concentration is sporadically high in Taiwan. Hg concentration is higher in the western coastal townships; Ni concentration is higher in the western and southeastern coastal townships. Pb and Zn concentrations have sporadic high values in Taiwan.

Table 1 shows the median concentrations of the eight soil heavy metals according to data of the 283 townships (N=283) of Taiwan in 1982–1986. The medians are: As 6.08 mg/kg, Cd 0.08 mg/kg, Cr 0.27 mg/kg, Cu 5.98 mg/kg, Pb 8.06 mg/kg, Hg 0.15 mg/kg, Ni 2.07 mg/kg, and Zn 10.17 mg/kg. Nearly all median metal levels are below standards set by TEPA and NYSDEC [28].

**Table 1:** **Township’s heavy metals contamination in soil during 1982-1986**^**a**^

**Heavy metals**	**1982-1986 (Township*N*** **= 283)**			**TEPA guidelines**		**NYSDEC guidelines**	
	**Q1**	**Median**	**Q3**	**Normal land-use**	**Agriculture land-use**	**Unrestricted use**	**Residential use**
As (mg/kg)	3.18	6.08	7.70	60	-	0.11	0.21
Cd (mg/kg)	0.03	0.08	0.15	20	5	0.43	0.86
Cr (mg/kg)^c^	< 0.01	0.27	1.23	250	-	29	58
Cu (mg/kg)	3.76	5.98	9.67	400	200	270	270
Pb (mg/kg)	6.25	8.09	11.06	2000	500	200	400
Hg (mg/kg)	0.09	0.15	0.23	20	5	-	-
Ni (mg/kg)	1.22	2.07	3.29	200	-	72	140
Zn (mg/kg)	7.35	10.17	14.12	2000	600	1100	2200

^a^*AS*, arsenic; *Cd*, cadmium; *Cr*, chromium; *Cu*, copper; *Hg*, mercury; *Ni*, nickel; *NYSDEC*, New York State Department of Environmental Conservation; *Pb*, lead; *TEPA*, Taiwan Environmental Protection Agency; *Zn*, zinc.

^c^Chromium NYSDEC guidelines value is the sum of the hexavalent and trivalent forms.

Table 2 shows the Spearman correlation coefficients among eight soil heavy metal concentrations in 1982–1986. Little correlation was observed between As and the other seven heavy metals (*r* = −0.17–0.18). The significantly positive correlations among the seven other metals imply that the townships in Taiwan are contaminated by several metals simultaneously.

**Table 2:** **Spearman correlation of heavy metals contamination in soil during 1982-1986**^**a**^

	**Cd**	**Cr**	**Cu**	**Hg**	**Ni**	**Pb**	**Zn**
As	0.18^*^	−0.03	−0.17^*^	0.09	0.14^*^	0.02	−0.01
Cd	1	0.34^*^	0.42^*^	0.34^*^	0.56^*^	0.27^*^	0.56^*^
Cr		1	0.24^*^	0.44^*^	0.33^*^	0.24^*^	0.35^*^
Cu			1	0.37^*^	0.46^*^	0.62^*^	0.65^*^
Hg				1	0.24^*^	0.37^*^	0.48^*^
Ni					1	0.21^*^	0.47^*^
Pb						1	0.50^*^

^a^*AS*, arsenic; *Cd*, cadmium; *Cr*, chromium; *Cu*, copper; *Hg*, mercury; *Ni*, nickel; *Pb*, lead; *Zn*, zinc.

^*^P < 0.05.

**Figure 2:**
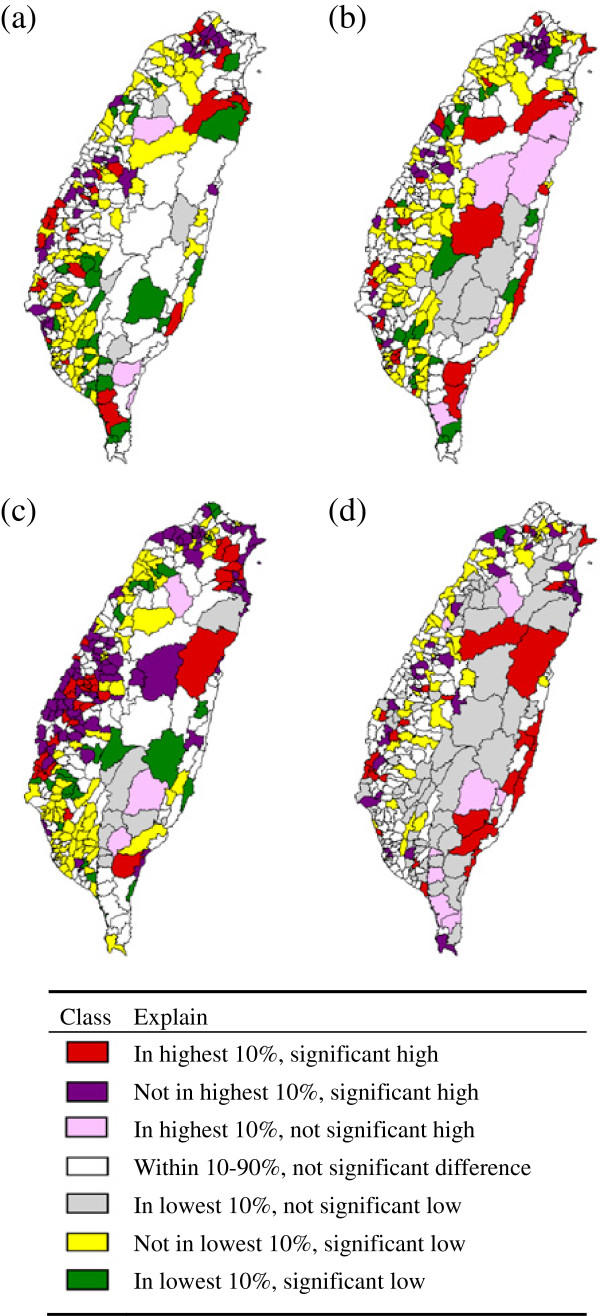
**Geographic patterns by lung cancer ASIR rank during 2001–2005.** (**a**) male lung AC, (**b**) female lung AC, (**c**) male lung SCC, (**d**) female lung SCC. Detailed legend: Geographic patterns by lung cancer ASIR rank of male/female above 30 years old during 2001–2005. (**a**) male lung AC, (**b**) female lung AC, (**c**) male lung SCC, (**d**) female lung SCC.

Figure 2 shows the geographical variations of lung AC and lung SCC incidence rates in males and females by rank in 2001–2005. The townships indicated by either red or purple denote that the incidence rates in those townships are significantly higher than the average incidence rate across Taiwan. Geographical variations were found to differ by cell type and gender. Figure 2a shows that male lung AC is clustered in Taipei and the western coastal area. Figure 2b shows that the female lung AC is clustered in Taipei and sporadically in the eastern, central, western and southern townships. Figure 2c shows the male lung SCC is clustered in the northern region, as well as in northeast coastal and central southwestern townships. Figure 2d shows that female lung SCC is clustered in the north east and the eastern coastal area, and sporadically in the southwestern coastal areas.

Table 3 shows the RRs of male lung AC and lung SCC in 2001–2005 in relation to specific metal concentration, based on the first quartile group of the soil heavy metal concentration as the reference group. The values of the reference groups of the eight metals are as follows: As 3.18 (mg/kg), Cd 0.03 (mg/kg), Cr0.01 (mg/kg), Cu 3.76 (mg/kg), Hg 0.09 (mg/kg), Ni 1.23 (mg/kg), Pb 6.25 (mg/kg), and Zn 7.36 (mg/kg). For male lung SCC, the townships where Cr>1.23 mg/kg have highly significant RR values (RR=1.49, 95% CI=1.37–1.61). The RR (95% CI) values of the townships exposed to Cu concentration levels at 3.76-5.98 mg/kg, 5.98-9.67 mg/kg, and>9.67 mg/kg are 1.10 (1.01-1.20), 1.19 (1.09–1.30), and 1.16 (1.06–1.28); all statistically significant. The RR values (95% CI) of the townships exposed to Hg from lower to higher levels are 1.22 (1.12–1.32), 1.27 (1.17–1.38), and 1.18 (1.08–1.29). The RR values of the townships exposed to Ni at >3.31 mg/kg (fourth quartile level) are significantly higher (RR=1.25, 95% CI=1.15–1.36). The RR values of the townships exposed to Pb at 6.25–8.08 mg/kg and 8.08–11.04 mg/kg are significantly higher: 1.17 (95% CI=1.07–1.27) and 1.15 (95% CI=1.06–1.25). The RR values of the townships exposed to Zn at 7.36–10.15 mg/kg, 10.15–14.02 mg/kg, and >14.02 mg/kg are significantly higher than those of the reference group: 1.12 (95% CI=1.02–1.22), 1.16 (95% CI = 1.06–1.27), and 1.18 (95% CI = 1.07–1.29). The result of the trend test for the male lung SCC incidence caused by exposure to Cr, Cu, Hg, Ni, and Zn indicates a statistical dose–response relationship. For male lung AC, only Cu and Ni show a significant dose–response relationship.

**Table 3:** **Township’s soil heavy metals concentration levels during 1982–1986 in relation to male lung cancer in 2001-2005**^**a,b**^

	**Lung AC**				**Lung SCC**			
**Heavy metals concentrations in soils (mg/kg)**	**Case/person year**	**RR**	**95% CI**	***P*-value**	**Case/person year**	**RR**	**95% CI**	***P*-value**
As								
>7.70	1,585/	0.95	0.89-1.02	0.18	1,385/	1.01	0.94-1.09	0.76
	5,260,215				5,260,215			
6.08 - 7.70	1,678/	0.90	0.84-0.96	<0.01	1,479/	0.98	0.91-1.06	0.61
	6,042,022				6,042,022			
3.18 - 6.08	1,722/	0.89	0.84-0.96	<0.01	1,443/	0.92	0.86-1.00	0.04
	6,324,337				6,324,337			
≦3.18	1,673/	1.00	-		1,329/	1.00	-	
	5,272,515				5,272,515			
*P* for trend				0.20				0.42
Cd								
>0.15	1,639/	1.05	0.97-1.14	0.26	1,463/	1.08	0.99-1.18	0.08
	5,515,399				5,515,399			
0.08 - 0.15	2,259/	1.03	0.96-1.11	0.37	1,756/	0.91	0.84-0.99	0.02
	7,725,756				7,725,756			
0.03 - 0.08	1,552/	0.97	0.9-1.04	0.36	1,380/	0.97	0.90-1.06	0.53
	5,602,900				5,602,900			
≦0.03	1,208/	1.00	-		1,037/	1.00	-	
	4,055,034				4,055,034			
*P* for trend				0.09				0.31
Cr								
>1.23	1,838/	1.02	0.94-1.10	0.66	1,780/	1.49	1.37-1.61	<0.01
	6,254,696				6,254,696			
0.27 - 1.23	1,667/	0.92	0.86-0.99	0.02	1,351/	1.00	0.93-1.09	0.95
	6,021,144				6,021,144			
0.01 - 0.27	1,373/	0.90	0.84-0.97	0.01	1,183/	1.04	0.96-1.13	0.34
	4,809,582				4,809,582			
≦0.01	1,780/	1.00	-		1,322/	1.00	-	
	5,813,668				5,813,668			
*P* for trend				0.76				<0.01
Cu								
>9.67	2,449/	1.18	1.09-1.29	<0.01	1,926/	1.16	1.06-1.28	<0.01
	8,119,166				8,119,166			
5.98 - 9.67	1,572/	1.10	1.02-1.19	0.02	1,397/	1.19	1.09-1.30	<0.01
	5,582,934				5,582,934			
3.76 - 5.98	1,432/	1.09	1.01-1.18	0.03	1,243/	1.10	1.01-1.20	0.03
	4,850,009				4,850,009			
≦3.76	1,205/	1.00	-		1,070/	1.00	-	
	4,346,981				4,346,981			
*P* for trend				<0.01				<0.01
Hg								
>0.23	2,034/	1.04	0.97-1.13	0.28	1,699/	1.18	1.08-1.29	<0.01
	7,226,678				7,226,678			
0.15 - 0.23	1,880/	1.05	0.98-1.13	0.18	1,684/	1.27	1.17-1.38	<0.01
	6,214,687				6,214,687			
0.09 - 0.15	1,599/	1.01	0.93-1.08	0.89	1,417/	1.22	1.12-1.32	<0.01
	5,539,629				5,539,629			
≦0.09	1,145/	1.00	-		836/	1.00	-	
	3,918,096				3,918,096			
*P* for trend				0.16				<0.01
Ni								
>3.31	1,745/	1.09	1.01-1.18	0.04	1,617/	1.25	1.15-1.36	<0.01
	6,010,486				6,010,486			
2.10 - 3.31	1,703/	1.04	0.97-1.11	0.31	1,357/	1.00	0.93-1.08	0.97
	5,818,529				5,818,529			
1.23 - 2.10	1,689/	0.95	0.89-1.02	0.14	1,365/	0.90	0.83-0.97	0.01
					5,870,834			
	5,870,834							
≦1.23	1,521/	1.00	-		1,297/	1.00	-	
	5,199,240				5,199,240			
*P* for trend				<0.01				<0.01
Pb								
>11.04	2,314/	1.03	0.96-1.11	0.41	1,854/	1.06	0.97-1.15	0.19
	7,943,003				7,943,003			
8.08 - 11.04	1,597/	1.01	0.94-1.09	0.79	1,465/	1.15	1.06-1.25	<0.01
	5,657,247				5,657,247			
6.25 - 8.08	1,467/	1.05	0.97-1.14	0.19	1,290/	1.17	1.07-1.27	<0.01
	4,827,411				4,827,411			
≦6.25	1,280/	1.00	-		1,027/	1.00	-	
	4,471,428				4,471,428			
*P* for trend				0.70				0.58
Zn								
>14.02	2,589/	1.09	1.00-1.19	0.05	2,122/	1.18	1.07-1.29	<0.01
	8,798,016				8,798,016			
10.15 - 14.02	1,781/	1.02	0.94-1.11	0.57	1,570/	1.16	1.06-1.27	<0.01
	6,351,742				6,351,742			
7.36 - 10.15	1,347/	1.06	0.97-1.15	0.20	1,152/	1.12	1.02-1.22	0.02
	4,519,372				4,519,372			
≦7.36	941/	1.00	-		792/	1.00	-	
	3,229,960				3,229,960			
*P* for trend				0.08				<0.01

^a^*AS*, arsenic; *Cd*, cadmium; *Cr*, chromium; *Cu*, copper; *Hg*, mercury; *Ni*, nickel; *Pb*, lead; *Zn*, zinc.

^b^Adjusted for age, each metals concentration in soil (e.g., As, Cd, Cr, Cu, Pb, Mg, Ni, and Zn).

Table 4 shows the RR values of female lung AC and lung SCC in 2001–2005 adjusted for sex and seven other continuous scales of metal concentration, based on the first quartile group of the soil heavy metal concentration as the reference group. Among eight soil heavy metals, the statistically significant finding for female SCC is that the RR is 1.38 (95% CI =1.14–1.68) when Cris0.27–1.23 mg/kg. The RR at the highest concentration levels are 1.29 (95% CI =1.04–1.60) and 1.29 (95% CI =1.03–1.62) for Ni and Zn, respectively. Those that reached a statistical significance in the dose–response relationship in the trend test are Cr (*P*=0.02), Ni (*P*=0.02), and Zn (*P*=0.02) for female lung SCC, and Cu (*P*< 0.01) and Zn (*P*=0.02) for female lung AC.

**Table 4:** **Township’s soil heavy metals concentration levels during 1982–1986 in relation to female lung cancer in 2001-2005**^**a,b**^

	**Lung AC**				**Lung SCC**			
**Heavy metals concentrations in soils (mg/kg)**	**Case/person year**	**RR**	**95% CI**	***P*-value**	**Case/person year**	**RR**	**95% CI**	***P*-value**
As								
>7.70	1,314/	0.92	0.85-1.00	0.04	243/	1.06	0.87-1.28	0.57
	4,959,803				4,959,803			
6.08 - 7.70	1,430/	0.92	0.86-1.00	0.04	217/	0.84	0.70-1.02	0.08
	5,752,167				5,752,167			
3.18 - 6.08	1,494/	0.94	0.87-1.01	0.12	209/	0.82	0.68-1.00	0.05
	6,139,517				6,139,517			
≦3.18	1,367/	1.00	-		222/	1.00	-	
	5,198,563				5,198,563			
*P* for trend				0.04				0.52
Cd								
>0.15	1,443/	1.05	0.96-1.15	0.26	222/	0.84	0.67-1.04	0.11
	5,285,544				5,285,544			
0.08 - 0.15	1,829/	1.05	0.97-1.14	0.23	295/	0.93	0.77-1.13	0.45
	7,645,830				7,645,830			
0.03 - 0.08	1,332/	1.03	0.95-1.12	0.47	188/	0.75	0.62-0.92	0.01
	5,303,029				5,303,029			
≦0.03	1,001/	1.00	-		186/	1.00	-	
	3,815,647				3,815,647			
*P* for trend				0.23				0.40
Cr								
>1.23	1,596/	0.97	0.89-1.06	0.50	252/	1.22	0.99-1.52	0.07
	6,126,512				6,126,512			
0.27 - 1.23	1,335/	0.92	0.85-0.99	0.03	253/	1.38	1.14-1.68	<0.01
	5,749,780				5,749,780			
0.01 - 0.27	1,141/	0.92	0.85-1.00	0.04	187/	1.19	0.97-1.47	0.09
	4,440,563				4,440,563			
≦0.01	1,533/	1.00	-		199/	1.00	-	
					5,733,195			
	5,733,195							
*P* for trend				0.40				0.02
Cu								
>9.67	2,145/	1.19	1.09-1.31	<0.01	331/	1.24	0.98-1.57	0.07
	8,174,628				8,174,628			
5.98 - 9.67	1,289/	1.06	0.97-1.16	0.17	202/	1.10	0.88-1.37	0.41
	5,340,797				5,340,797			
3.76 - 5.98	1,181/	1.09	1.00-1.19	0.06	199/	1.16	0.94-1.44	0.17
	4,511,228				4,511,228			
≦3.76	990/	1.00	-		159/	1.00	-	
	4,023,397				4,023,397			
*P* for trend				<0.01				0.12
Hg								
>0.23	1,696/	0.91	0.83-0.99	0.02	264/	1.00	0.80-1.24	0.98
	7,154,506				7,154,506			
0.15 - 0.23	1,548/	0.92	0.85-1.00	0.04	252/	1.03	0.84-1.27	0.78
	6,049,340				6,049,340			
0.09 - 0.15	1,316/	0.93	0.86-1.01	0.09	224/	1.08	0.88-1.33	0.45
	5,151,875				5,151,875			
≦0.09	1,045/	1.00	-		151/	1.00	-	
	3,694,329				3,694,329			
*P* for trend				0.03				0.80
Ni								
>3.31	1,532/	1.09	1.00-1.19	0.04	262/	1.29	1.04-1.60	0.02
	5,888,186				5,888,186			
2.10 - 3.31	1,401/	1.05	0.97-1.14	0.21	219/	1.07	0.88-1.31	0.48
	5,546,267				5,546,267			
1.23 - 2.10	1,523/	1.07	0.99-1.15	0.11	230/	1.01	0.83-1.23	0.90
	5,764,114				5,764,114			
≦1.23	1,149/	1.00	-		180/	1.00	-	
	4,851,483				4,851,483			
*P* for trend				0.07				0.02
Pb								
>11.04	2,003/	0.99	0.92-1.07	0.80	296/	1.05	0.86-1.29	0.61
	7,927,859				7,927,859			
8.08 - 11.04	1,294/	0.94	0.87-1.02	0.15	234/	1.21	0.98-1.49	0.07
	5,333,266				5,333,266			
6.25 - 8.08	1,155/	0.93	0.85-1.01	0.07	195/	1.09	0.88-1.35	0.42
	4,578,886				4,578,886			
≦6.25	1,153/	1.00	-		166/	1.00	-	
	4,210,039				4,210,039			
*P* for trend				0.84				0.58
Zn								
>14.02	2,193/	1.11	1.01-1.22	0.02	347/	1.29	1.03-1.62	0.03
	8,807,482				8,807,482			
10.15 - 14.02	1,538/	1.09	0.99-1.19	0.07	243/	1.09	0.87-1.37	0.45
	6,137,430				6,137,430			
7.36 - 10.15	1,098/	1.05	0.96-1.15	0.28	178/	1.09	0.86-1.37	0.48
	4,196,903				4,196,903			
≦7.36	776/	1.00	-		123/	1.00	-	
	2,908,236				2,908,236			
*P* for trend				0.02				0.02

^a^*AS*, arsenic; *Cd*, cadmium; *Cr*, chromium; *Cu*, copper; *Hg*, mercury; *Ni*, nickel; *Pb*, lead; *Zn*, zinc.

^b^Adjusted for age, each metals concentration in soil (e.g., As, Cd, Cr, Cu, Pb, Mg, Ni, and Zn).

## Discussion

The latent period of lung cancer incidence caused by environmental risk factors has been estimated at 15 years [22]. Hence, we chose to compare soil heavy metal concentration during1982–1986 with 2001–2005 data for lung cancer incidence.

However, because lung cancer is a multiple risk factor cancer with a longer latent period, it is difficult to establish an association between lung cancer and soil heavy metal contamination. Certainly, regional differences in smoking prevalence must be considered. However, according to a smoking prevalence survey in Taiwan [29], smoking prevalence doesn’t cluster in accordance with lung cancer incidences for either men or women. Since smoking behavior may not explain the clustering, the potential for environmental risk factors may exist. An important finding of this study is that, for some heavy metals, soil concentrations at levels lower than regulatory standards appear to be associated with lung cancer incidence. Our results indicating lower levels of soil heavy metal contamination still show a significant dose–response relationship between lung cancer incidence and some soil heavy metal contaminations. For example, Tables 3 and 4 shows that Cr, Ni, and Zn in both males and females, and Cu and Hg in males, have significant dose–response relationships with lung SCC. Cu in both males and females, Ni in males, and Zn in females have significant dose–response relationships with lung AC.

Previous studies have found that exposure to higher Cr concentrations in the workplace has a positive correlation with lung cancer. However, these studies have mainly investigated factory workers exposed to higher doses [14,30-32]. Our study targeted the general population rather than factory workers, and found that the Cr concentration in soil is relatively lower than that of the TEPA guideline. Beveridge et al. [33] conducted a population-based case control study to explore the correlation between Cr and lung cancer, and determined no significant correlation. In fact, Beveridge et al. [33] did not distinguish the cell-type-specific pathologies of lung cancer for analysis. By adopting an ecological study design, we found that soil Cr levels were associated with lung SCC, but not with lung AC.

This study found that Cu concentration in soil has a significantly positive correlation with lung AC incidence risk for both sexes and with lung SCC for males, thus suggesting a dose–response relationship. Previous studies have indicated that the ceruloplasmin concentration in the serum of lung cancer patients has been higher than those of control groups [34,35], and the Cu/Zn ratio in the serum of lung cancer patients has also been higher [20]. Other studies have reported that the Cu in cancer patients has not been significantly different from those of control groups, and the Cu concentration in hair has been significantly lower than those of control groups [36]. Our population-based ecological study revealed that exposure to soil copper has a positive dose–response relationship with lung AC for both sexes and with lung SCC for males.

Numerous studies have indicated that exposure to Ni increases the incidence risk of lung cancer [33,37,38]; however, some studies have reported an opposite finding [39-41]. These studies have not differentiated the cell-type-specific pathologies of lung cancer. A study examining heavy metal accumulation in lung tissue of lung cancer patients showed that the Ni concentration of the lung cancer patients was higher than that of the control group [42,43]. Sunderman et al. [44] indicated that more lung SCC cases and fewer lung AC cases are found in nickel factory workers than in the normal population. However, Sunderman et al. [44] believed that their study may have encountered a selection bias. Kuo et al. [42] conducted a case control study in Taiwan and found that the lung tissues of cancer patients had a higher Ni concentration in lung biopsy specimens than in those of the control group, and the Ni concentrations of lung AC and lung SCC patients did not reach a statistical difference. This indicates that Ni may cause either lung AC or lung SCC. Our study included nearly all lung cancer cases in Taiwan; thus, the selection bias could be minimized. We not only found a dose–response relationship of Ni with lung SCC in both sexes and with lung AC in males but also observed a higher propensity for Ni to be associated with lung SCC than with lung AC, based on RR values.

Most previous studies have determined that a deficiency of Zn increases the incidence risk of several cancers [45,46]. Zn is an essential trace element in organisms, and is critical in the stabilization of cell membranes [47,48]. A cohort study in the United States found that males who ingest more than 100 mg of Zn per day may have a higher incidence risk (RR = 2.29) of prostate cancer than those who do not ingest Zn; and ingestion of Zn for more than 10 years has a higher incidence risk of prostate cancer (RR = 2.37) [49]. Our study found that the soil Zn concentration in the surveyed townships has a dose–response relationship with male and female lung SCC. This finding is consistent with that of Coyle et al. [22] regarding Zn discharge and lung cancer in the investigated area. Díez et al. [20] found that the serum Zn concentration of lung cancer patients was lower than that of the control group, in contrast to the results of the current study. Thus, the correlation between Zn and cancer should be studied further.

The data on concentrations of air pollutants during 1994–1998, acquired from the Taiwan Air Quality Monitoring Network operated by the TEPA, were also introduced into the analyses. (data not shown) Only 48 townships in these data possessed records of soil heavy metal concentrations. The mean concentrations of CO, NO, NO2, O3, PM10, and SO2 among these 48 townships were used to adjust the regression model. The medians (quartiles Q1-Q3) of these air pollutants are CO 0.61 ppm (0.53–0.82), NO 6.82 ppb (4.28–10.35), NO2 20.43 ppb (16.40–25.26), O3 22.37 ppb (20.76–25.66), PM10 62.89 μg/m^3^ (50.06–77.00), and SO2 5.34 ppb (3.31–6.60). After applying the adjustment for air pollutants to the analyses, RR trend test results lost statistical significance in the following analyses: lung AC and Cu, Ni in males, lung AC and Zn in females, lung SCC and Cu, Hg, in males, and lung SCC and Cr, Ni, Zn in females. Statistical significance was exhibited only for lung AC and Cu in females, and lung SCC and Cr, Ni, Zn in males. Although this change may be due to a reduced statistical power, stronger relationships between lung AC and Cu in females and between lung SCC and Cr, Ni, and Zn in males were established. However, the air pollution monitoring indicators sourced from 1994–1998 data and the soil heavy metals in 1982–1986 exhibit a time lag. After we merged the data of air pollutants and soil heavy metal concentrations, only the data from 48 townships remained, which may have reduced the statistical power.

This study has the following limitations. First, it is ecological and subject to the ecological fallacy, since confounding factors of individuals cannot be adjusted for and regional metal concentrations may not reflect individual exposure levels. Second, this study examined soil heavy metal concentrations, but did not address the effects of different forms of heavy metals, nor address the exposure pathways in the body (e.g., breathing or ingestion). Third, we attempted to make adjustments by using smoking prevalence in the townships; however, the smoking prevalence data are from the 2001 NHIS database. Because of this, we were not able to account adequately for the influence of any regional differences in smoking prevalence upon our results.

## Conclusion

The current study suggests that a higher Cr concentration in soil is associated with male and female lung SCC; a higher soil Cu concentration is associated with male and female lung AC and lung SCC; a higher soil Ni concentration is associated with male lung AC, and male and female lung SCC; and a higher soil Zn concentration is associated with female lung AC and male and female lung SCC. This study determined that a dose–response relationship may exist between low-dose soil heavy metal concentration and lung cancer incidence according to specific cell- type; however, the relevant mechanism should be explored further.

## Abbreviations

AC: Adenocarcinoma; AS: Arsenic; ASIR: Age-standardized incidence rate; Cd: Cadmium; CI: Confidence interval; Cr: Chromium; Cu: Copper; GIS: Geographical information system; Hg: Mercury; ICD-O: International classification of diseases for oncology; NCRP: National cancer registration program; Ni: Nickel; NYSDEC: New York state department of environmental conservation; Pb: Lead; RR: Relative ratio; SCC: Squamous cell carcinoma; TEPA: Taiwan environmental protection agency; Zn: Zinc.

## Competing interests

The authors declare that they have no competing interests.

## Authors’ contributions

LYP participated in the design and conducted the study, interpreted the results, and helped draft and edit the manuscript. HHH participated in the design, conducted the study, and contributed to the writing of the manuscript, HJY and LCC participated in the design, conducted the study, and contributed to the writing of the revised manuscript. HCC and WCL assisted in conducting the study. SYH helped revise the manuscript. KPC, CSC, and SSY participated in the data analysis. All of the authors read and approved the final manuscript.

## Pre-publication history

The pre-publication history for this paper can be accessed here:

http://www.biomedcentral.com/1471-2458/13/330/prepub
